# Sea urchin larvae utilize light for regulating the pyloric opening

**DOI:** 10.1186/s12915-021-00999-1

**Published:** 2021-04-06

**Authors:** Junko Yaguchi, Shunsuke Yaguchi

**Affiliations:** 1 Shimoda Marine Research Center, University of Tsukuba, 5-10-1 Shimoda, Shizuoka, 415-0025 Japan; 2 PRESTO, JST, 4-1-8 Honcho, Kawaguchi, 332-0012 Japan

**Keywords:** Sea urchin, Opsin, Serotonin, Nitric oxide, Gut

## Abstract

**Background:**

Light is essential for various biological activities. In particular, visual information through eyes or eyespots is very important for most of animals, and thus, the functions and developmental mechanisms of visual systems have been well studied to date. In addition, light-dependent non-visual systems expressing photoreceptor Opsins have been used to study the effects of light on diverse animal behaviors. However, it remains unclear how light-dependent systems were acquired and diversified during deuterostome evolution due to an almost complete lack of knowledge on the light-response signaling pathway in Ambulacraria, one of the major groups of deuterostomes and a sister group of chordates.

**Results:**

Here, we show that sea urchin larvae utilize light for digestive tract activity. We found that photoirradiation of larvae induces pyloric opening even without addition of food stimuli. Micro-surgical and knockdown experiments revealed that this stimulating light is received and mediated by Go(/RGR)-Opsin (Opsin3.2 in sea urchin genomes) cells around the anterior neuroectoderm. Furthermore, we found that the anterior neuroectodermal serotoninergic neurons near Go-Opsin-expressing cells are essential for mediating light stimuli-induced nitric oxide (NO) release at the pylorus. Our results demonstrate that the light>Go-Opsin>serotonin>NO pathway functions in pyloric opening during larval stages.

**Conclusions:**

The results shown here will lead us to understand how light-dependent systems of pyloric opening functioning via neurotransmitters were acquired and established during animal evolution. Based on the similarity of nervous system patterns and the gut proportions among Ambulacraria, we suggest the light>pyloric opening pathway may be conserved in the clade, although the light signaling pathway has so far not been reported in other members of the group. In light of brain-gut interactions previously found in vertebrates, we speculate that one primitive function of anterior neuroectodermal neurons (brain neurons) may have been to regulate the function of the digestive tract in the common ancestor of deuterostomes. Given that food consumption and nutrient absorption are essential for animals, the acquirement and development of brain-based sophisticated gut regulatory system might have been important for deuterostome evolution.

## Background

Light plays crucial roles in biological processes such as photosynthesis and vision. Because visual systems involving eyes are very important for animal behaviors, a number of previous studies have investigated how the integrated circuits that mediate light stimulus develop and function [1–5]. In addition, recent studies have suggested that non-visual systems dependent on light also play essential roles in the life activities of animal, such as circadian rhythms [6, 7]. Many of these light-dependent systems rely on photoreceptor Opsin members, which belong to the group of sensory G-protein-coupled receptors (GPCRs), and their functional diversity has led us to consider how visual/non-visual systems developed during evolution to utilize light as an external signaling source [8–10]. In addition, structural and molecular analyses of Opsins in invertebrates such as tunicates and jellyfish have led us to consider the evolution of photoreceptor proteins [11, 12]. However, it is still difficult to precisely compare the functions of and predict the evolution of the light-dependent system in deuterostomes because we do not have experimental data about the precise function of the Opsin family in Ambulacraria, a sister group of chordates, although the evolutionary comparisons based on the primary structures have been performed [13].

Sea urchins are echinoderms and members of one of the phyla of Ambulacraria, and their embryos/larvae have been used as model organisms in developmental and cell biology for more than a century, but scientifically reproducible light-response data from embryos/larvae under genetic modifications have never been reported thus far, although they are under debate [13–15], and it has been reported that Ambulacrarian adults, including sea urchins, express a wide variety of Opsins and show light-response behaviors [16–18]. Even so, none of the Opsin family functions has been reported, although the presence of 6 Opsin genes (Opsin1, 2, 3.1, 3.2, 4, and 5) has been identified in the genomes of *Strongylocentrotus purpuratus* [13, 19] and *Hemicentrotus pulcherrimus* [20] and that the diversity and evolution of the Opsin family based on their gene structures were well discussed [21]. In addition, the functions of sea urchin nervous systems, which are presumptive mediators between photoreception and larval/adult behaviors, have been reported only in a limited number of papers. For example, serotonergic neurons, which are present exclusively in the anterior neuroectoderm, are required for gravity-dependent swimming behaviors [22], and a nitric oxide (NO) neuron in the pylorus regulates the pyloric opening [23]. On the other hand, a functional analysis of ciliary band neurons, including recently identified cholinergic neurons [24], has never been performed [25], and we do not have any experimental data about the relationship between the nervous system and light stimuli in sea urchins. In this study, we report that the sea urchins utilize light for regulating their pyloric opening with the function of serotonergic neurons and nitric oxide. Because the serotonergic neurons and Go-Opsin-expressing cells are present in/near the larval anterior neuroectoderm [26, 27], which is suggested as developmentally homologous to chordate brains [28–30], these data show the clear evidence for the presence of light-dependent brain-gut regulatory system in Ambulacraria and suggest that one of the primitive functions of deuterostome brains is regulating the digestive tract function.

## Results

**Fig. 1:**
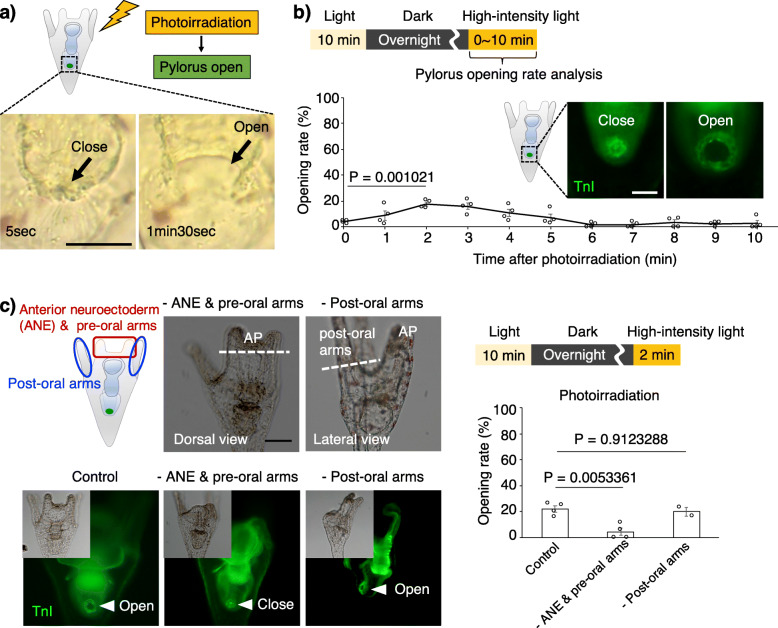
Photoirradiation opens the pylorus in sea urchin larvae. **a** Photoirradiation drives the pyloric opening. Images of 5 s and 1 min 30 s after photoirradiation were captured from Supplementary Movie 1. **b** The set light condition was 10 min of light, overnight in darkness, and photoirradiation by high-intensity light. The graph shows the pyloric opening rates between 0 and 10 min after photoirradiation. The fluorescence images show pyloric closing (left) and opening (right), as visualized by TnI immunohistochemistry. *N* = 4; *n* (0 min) = 60, 39, 29, 43; *n* (1 min) = 45, 21, 30, 32; *n* (2 min) = 45, 26, 33, 54; *n* (3 min) = 43, 28, 23, 31; *n* (4 min) = 38, 28, 37, 24; *n* (5 min) = 68, 39, 35, 37; *n* (6 min) = 41, 28, 40, 26; *n* (7 min) = 72, 27, 23, 39; *n* (8 min) = 43, 27, 31, 13; *n* (9 min) = 84, 29, 29, 29; *n* (10 min) = 76, 38, 24, 21. The increase in the opening rate between 0 and 2 min after photoirradiation was assessed by the Welch two-sample *t*-test. Error bars show SE. More than 40% of larvae opened their pylorus in response to the photoirradiation (= sum of open rate from 1 to 5 min (8.9% (1 min) + 17.9% (2 min) + 16.1% (3 min) + 10.9% (4 min) + 7.0% (5 min)) was divided by 1.5 min (average open time)). **c** The region around the anterior neuroectoderm was necessary for pyloric opening induced by photoirradiation. The images in the upper row show the location at which the anterior neuroectoderm and pre-oral arms (− ANE and pre-oral arms) and post-oral arms (− post-oral arms) were removed. The lower images show open and closed pylori 2 min after photoirradiation. The inset of each image shows the bright-field images. The graph shows the opening rate of the pylorus in control larvae, larvae without the AP and pre-oral arms, and larvae without post-oral arms 2 min after photoirradiation. *N* = 2–4; *n* (control) = 19, 20, 24, 45; *n* (ANE and pre-oral arms less) = 13, 15, 17, 17; *n* (post-oral arms less) = 13, 12. We used one-way ANOVA followed by Tukey’s post hoc test. Error bars show SE. Scale bars in **a** and **b** = 20 μm and in **c** = 50 μm

To investigate the responses of sea urchins to light, we observed the living larvae of *H. pulcherrimus*, which were transferred to a bright field just after incubation under dark conditions overnight, under a microscope for several minutes. As a result, we discovered that the pylori of some larvae opened in response to photoirradiation (Fig. 1a, Additional file 1: Fig. S1, Additional file 2: Movie 1). Since the opening time and frequency were variable depending on the incubation conditions prior to the light stimulus (Additional file 1: Fig. S2a, b), to estimate the opening ratio precisely, we set a constant light/dark cycle (Fig. 1b; 10 min of light, 16 h of dark, 0–10 min of photoirradiation [photon flux density, 1000 μmol m^−2^ s^−1^]) and checked pyloric opening/closing with immunohistochemistry using anti-TroponinI, which detects the pyloric sphincter [31], in the fixed larvae. The ratio of pyloric opening/closing events in all the subsequent experiments was measured in fixed and immunostained larvae (see the “Methods” section). The pylori of approximately 20% of the larval population were opened 2 min after photoirradiation, and by 6 min, most of them were closed under the set light conditions. The average pyloric opening rates were 4.0% (0 min), 8.9% (1 min), 17.9% (2 min), 16.1% (3 min), 10.9% (4 min), 7.0% (5 min), 1.5% (6 min), 1.7% (7 min), 3.4% (8 min), 2.2% (9 min), and 2.7% (10 min). The average pyloric opening time among individuals was approximately 1.5 min (*n* = 8, Additional file 1: Fig. S1), and the pylori of more than 40% of the larval population opened in response to the light stimulus (Fig. 1b; see the figure legend for the calculation). The reason why only 40% of the larvae responded to light will be elucidated in the future. This ratio jumped to more than 80% with food (mono-cellular algae) (Additional file 1: Fig. S3), indicating that food stimulation represents another regulatory pathway that activates the digestive tract, although we did not focus on this pathway in this paper. On the other hand, the timing of the response did not vary with or without food, suggesting that the light-dependent regulatory pathway that mediates pyloric opening is very stable. Based on these observations, the light>pylorus pathway is present in sea urchin larvae. We checked pyloric opening/closing 2 min after photoirradiation following 10 min of light and 16 h of dark in all of the following experiments (Fig. 1b).

**Fig. 2:**
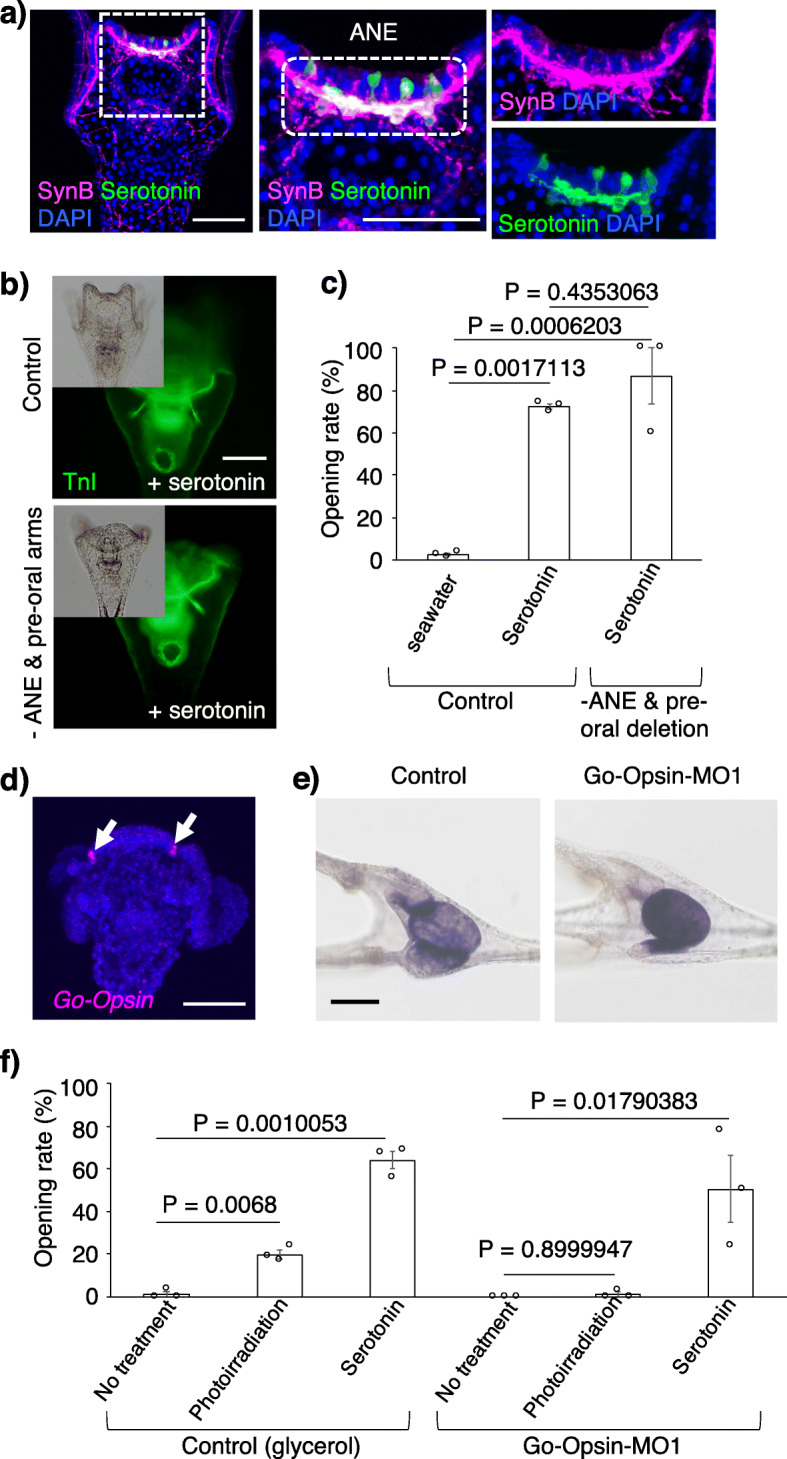
The anterior neuroectoderm plays a crucial role in opening the pylorus. **a** Serotonergic neurons and pan-neurons recognized by Synaptotagmin B (synB) in sea urchin larvae. Green, serotonin; magenta, SynB; blue (DAPI), nuclei. **b** Serotonin induced pyloric opening in both control larvae and larvae without the ANE and pre-oral arms. The inset of each image shows the bright-field images. **c** The graph shows the opening rate of the pylorus in control larvae treated with seawater, in control larvae 2 min after the addition of serotonin, and in larvae without the ANE and pre-oral arms 2 min after the addition of serotonin. *N* = 3; *n* (with seawater) = 55, 79, 74; *n* (with serotonin) = 23, 11, 10; *n* (without the ANE and pre-oral arms and with serotonin) = 5, 8, 4. Error bars show SE. **d** The expression pattern of *Go-Opsin* (*opn3.2*) in *Hemicentrotus pulcherrimus* (arrows). **e** The activity of alkaline phosphatase in the gut was invariant in control and Go-Opsin morphants. **f** The graph shows the opening rate of the pylorus in control larvae and Go-Opsin morphants 2 min after photoirradiation or the addition of serotonin. *N* = 3; *n* (control; no treatment) = 45, 28, 22; *n* (control; photoirradiation) = 78, 63, 54; *n* (control; +serotonin) = 25, 49, 38; *n* (Go-Opsin morphants with no treatment) = 16, 14, 14; *n* (Go-Opsin morphants with photoirradiation) = 33, 32, 11; *n* (Go-Opsin morphants with serotonin) = 9, 51, 28. Error bars show SE. Scale bars in **a**, **b**, **d**, and **e** = 50 μm

It is reasonably expected that this light>pylorus pathway is managed by the nervous and photoreception systems, which are intensively present in the anterior region of the larva [26, 32]. To examine which body part plays the central role in the light>pylorus pathway, we initially removed the anterior neuroectoderm/pre-oral arms or post-oral arms and checked the pyloric opening/closing under photoirradiation (Fig. 1c). The average pyloric opening rates were 21.7% (control), 4.4% (without ANE and pre-oral arms), and 19.9% (without post-oral arms). The pylori in larvae without anterior neuroectoderm/pre-oral arms did not open, whereas they did open in larvae without post-oral arms, indicating that the anterior neuroectoderm/pre-oral arms are necessary for this pathway (Fig. 1c). Within the anterior neuroectoderm and the adjacent regions, serotonergic neurons and Go-Opsin-expressing cells, respectively, are exclusively present [26, 32] (Fig. 2a, d). Therefore, we examined whether these cells are involved in the light>pylorus pathway. First, we applied serotonin and checked pyloric opening/closing without photoirradiation. Intriguingly, the pylori of serotonin+ larvae opened in a concentration-dependent manner, even without photoirradiation, whereas pylori of the seawater-applied larvae did not (Fig. 2c, Additional file 1: Fig. S4a, b). In addition, serotonin opened the pylori of larvae without anterior neuroectoderm/pre-oral arms (Fig. 2b, c). The average pyloric opening rates were 2.5% (+seawater), 72.2% (+serotonin), and 86.7% (without the ANE and pre-oral arms +serotonin). In contrast, in tryptophan 5-hydroxylase (TPH; serotonin synthase) morphants (in which the TPH function is knock downed using a specific morpholino), the pylorus did not open under photoirradiation (Additional file 1: Fig. S4c-e). These data suggest that serotonin, which is produced exclusively in the anterior neuroectoderm, is essential for the mechanisms of pyloric opening under photoirradiation in sea urchin larvae. Next, we knocked down the function of Go-Opsin, which is expressed in neurons adjacent to the anterior neuroectoderm in *H. pulcherrimus,* as shown in *S. purpuratus* [26, 27] (Fig. 2d, Additional file 1: Fig. S5), with morpholino oligos, because the other members of the Opsin family are not expressed around this region during the larval stages [13]. In Go-Opsin morphants, in which endoderm activity seemed to be normal (Fig. 2e), the pylorus did not open even under photoirradiation, but serotonin rescued this effect (Fig. 2f, Additional file 1: Fig. S6). The average pyloric opening rates were 1.2% (control; no treatment), 19.7% (control; photoirradiation), 63.9% (control; +serotonin), 0% (Go-Opsin morphants with no treatment), 1.0% (Go-Opsin morphants with photoirradiation), and 50.4% (Go-Opsin morphants with serotonin). This suggests that serotonin functions in pyloric opening downstream of the light>Go-Opsin pathway. It is very intriguing that the timing of the peak of pyloric opening in serotonin-supplied larvae was the same as that in photoirradiated larvae (Additional file 1: Fig. S4); this supports the idea that serotonin functions downstream of photoirradiation.

**Fig. 3:**
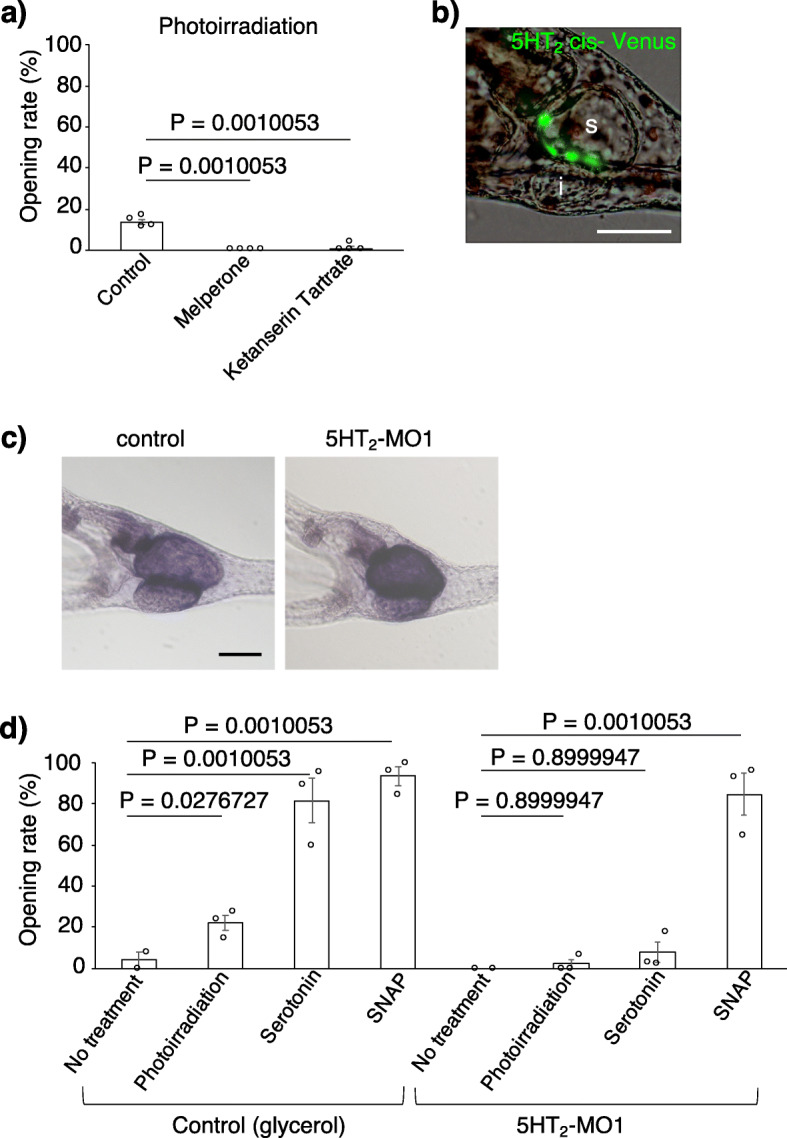
The 5HT_2_ receptor mediates the light>pylorus signaling pathway. **a** The opening rate of the pylorus under photoirradiation was extremely reduced by the addition of melperone hydrochloride and ketanserin tartrate (5-HT_2_ receptor antagonists). *N* = 4; *n* (control) = 54, 26, 42, 66; *n* (melperone hydrochloride) = 15, 17, 27, 36; *n* (ketanserin tartrate) = 17, 17, 27, 25. Error bars show SE. **b** The putative *cis*-regulatory elements of the 5HT_2_ receptor drove Venus signaling in the stomach. The rate of Venus expression in the stomach was 81.4% (57/70) in all larvae that had Venus signals. s, stomach; i, intestine. **c** The activity of alkaline phosphatase in the gut was invariant in control and 5HT_2_ morphants. **d** The graph shows that the 5HT_2_ receptor was required for the light>pylorus signaling pathway. *N* = 2–3; *n* (control with no treatment) = 25, 37; *n* (control with photoirradiation) = 21, 11, 55; *n* (control with serotonin) = 25, 19, 22; *n* (control with SNAP) = 28, 14, 19; *n* (5HT_2_ morphants with no treatment) = 13, 16; *n* (5HT_2_ morphants with photoirradiation) = 16, 16, 17; *n* (5HT_2_ morphants with serotonin) = 35, 45, 23; *n* (5HT_2_ morphants with SNAP) = 28, 28, 37. Error bars show SE. Scale bars in **b** and **c** = 50 μm

**Fig. 4.**
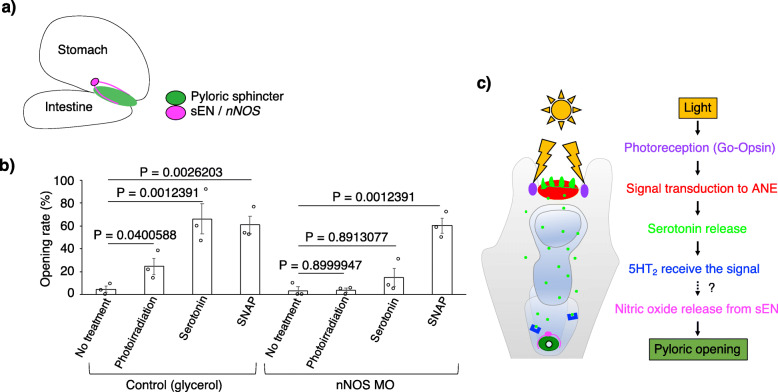
The light>serotonin pathway activates enteric neurons in the stomach (sENs), which express *nNOS* and induce pyloric opening. **a** A schematic image of *neuronal nitric oxide synthase* (*nNOS*)-expressing enteric neurons (sENs; magenta) with axon-like structures around the pyloric sphincter (green) [23]. **b** The graph shows the pyloric opening rate in control and nNOS morphants under photoirradiation, the addition of serotonin, and the addition of SNAP. *N* = 3; *n* (control with no treatment) = 10, 21, 26; *n* (control with photoirradiation) = 14, 21, 28; *n* (control with serotonin) = 15, 12, 30; *n* (control with SNAP) = 21, 19, 13; *n* (nNOS morphants with no treatment) = 13, 11, 10; *n* (nNOS morphants with photoirradiation) = 21, 17, 20; *n* (nNOS morphants with serotonin) = 19, 26, 12; *n* (nNOS morphants with SNAP) = 63, 12, 29. Error bars show SE. **c** A schematic diagram of the light>pylorus signaling pathway in sea urchin larvae

Because the sea urchin neurons do not tend to form synaptic structures in a manner similar to serotonergic neurons in mammalian brains [33, 34], it is expected that serotonin, which is secreted from the anterior neuroectoderm, will be dispersed through the entire body of the larvae and activate locally present receptors. To examine how the serotonin pathway regulates the pylorus, we pharmacologically inhibited serotonin receptors and checked pyloric opening/closing. When the wide-ranging monoamine/serotonin receptor inhibitors, methysergide maleate [35] and asenapine maleate [36], were applied, the pylorus did not open even under photoirradiation (Additional file 1: Fig. S7a). In sea urchin genomes, 4 types of serotonin receptors, 5HT_1_, 5HT_2_, 5HT_4/5/6_, and 5HT_7_, were identified, and their phylogenetic positions among bilaterians were confirmed [19, 20, 37]. Because 5HT_2_ and 5HT_7_ are mainly expressed during the embryonic and larval stages based on their temporal expression patterns [38, 39] and because methysergide maleate works as an agonist of 5HT_1_ in mammals, we inhibited 5HT_2_ and 5HT_7_ with their specific antagonists, melperone [40] and ketanserin tartrate [40] (for 5HT_2_) and SB269970 [41] (for 5HT_7_). The results suggest that 5HT_2_, but not 5HT_7_, is involved in the serotonin>pylorus pathway under photoirradiation (Fig. 3a, Additional file 1: Fig. S7a, b). The average pyloric opening rates were 13.8% (control), 0% (melperone hydrochloride), and 1.0% (ketanserin tartrate). In addition, when the 5HT_2_ receptor was knocked down (Fig. 3c), the pylori of the morphants responded to neither photoirradiation nor serotonin but did respond to the nitric oxide (NO) donor S-nitroso-N-acetyl-D,L-penicillamine (SNAP) [23] (Fig. 3d, Additional file 1: Fig. S7d). This SNAP experiment was performed because we had recently reported that NO is involved in the pyloric opening in sea urchin larvae, similar to mammals [23]. The average pyloric opening rates were 4% (control with no treatment), 21.9% (control with photoirradiation), 81.6% (control with serotonin), 93.5% (control with SNAP), 0% (5HT_2_ morphants with no treatment), 2.1% (5HT_2_ morphants with photoirradiation), 7.5% (5HT_2_ morphants with serotonin), and 84.7% (5HT_2_ morphants with SNAP). These data suggest that 5HT_2_ mediates the pathway between serotonin and NO-dependent pyloric opening. In situ hybridization did not detect the expression of 5HT_2_ mRNA in our hands, but the microinjection of a DNA reporter construct, in which the putative *cis*-regulatory elements of 5HT_2_ and DNA encoding fluorescent protein Venus were fused, drove the expression of the reporter Venus in the stomach (Fig. 3b, and Additional file 1: Fig. S8; see the “Methods” section [microinjection of morpholino anti-sense oligonucleotides (MO), mRNAs, and DNA]), suggesting that 5HT_2_ is expressed in the stomach. Because the Venus signal representing 5HT_2_ expression did not localize to the pylorus but localized everywhere in the stomach, it is likely that serotonin from the anterior neuroectoderm indirectly activates the NO enteric neurons (hereafter referred to as sEN [23]; Fig. 4a) and the pyloric sphincter. To confirm whether the sEN functions in the pathway between photoirradiation and pyloric opening, we knocked down neuronal nitric oxide synthase (nNOS), which is expressed in the sEN, and checked pyloric opening/closing. When nNOS was attenuated, neither photoirradiation nor exogenous serotonin opened the pylorus, whereas SNAP did, indicating that NO release from the sEN is essential for opening the pylorus downstream of the light>serotonin pathway (Fig. 4b). The average pyloric opening rates were 4.5% (control with no treatment), 24.6% (control with photoirradiation), 66.1% (control with serotonin), 60.9% (control with SNAP), 3.3% (nNOS morphants with no treatment), 3.6% (nNOS morphants with photoirradiation), 14.8% (nNOS morphants with serotonin), and 60.4% (nNOS morphants with SNAP).

## Discussion

Taken together, our data suggest that sea urchin larvae utilize light to open the pylorus through the photoirradiation>Go-Opsin>anterior neuroectoderm>serotonin>5HT_2_>sEN/NO>pylorus pathway (Fig. 4c). Two points shown here are especially intriguing: (1) light behaves as a tool to regulate the activity of the digestive tract, and (2) the light-dependent signaling pathway in echinoderm larvae was revealed under the modification of genetic function. Because neither stable pigmented shade nor sophisticated neural integration was observed around Go-Opsin-expressing cells, the photoreceptors of sea urchin larvae work as a non-visual and non-directional system [26]. Light stimulates several non-visual activities in a wide variety of organisms, as a number of previous scientific works have proven. For example, non-visual Opsin3, which is expressed in human epidermal melanocytes, mediates pigmentation [42], and Opsin5, which is expressed in the bird brain, is essential for seasonal reproduction [6]. In sea urchin larvae, light, which is received around the anterior neuroectoderm, likely drives digestive activity. Although it has been well reported that vertebrates utilize light-dark cycles, i.e., circadian rhythms, for their digestive activities, such as gut motility, gene expression, and mucosal production [43, 44], these activities are relatively slow since they change over a 24-h cycle. In addition, to drive established regulatory pathways, individuals need to prepare the execution unit, such as the photoreception system, products of clock genes, signal receivers in the gut, and mediators between each. Compared with these pathways, what we describe here happens within a relatively short time during early developmental stages when embryos/larvae are still establishing tissues and organs and requires only a simple signaling pathway involving a few factors, meaning that this phenomenon is likely a reflexive movement and not a circadian rhythm. In contrast, compared with the conventional light reflex carried out by the combination of photoreceptors, neurons, and muscles in many kinds of animals, including their embryos/larvae [45], pyloric opening is relatively slow. It would be of interest to see the timescale of each response step of the light > pylorus pathway in the future.

This reflex seems to be important not only for gut function but also for gut development. It is well known that the precise repetitive contraction and relaxation of the enteric muscle is essential for its development [46, 47]. Because sea urchin larvae have to swallow and digest algae from the beginning of their larval lives and because, during this period, there is no liquid or baby food such as milk to induce the maturation of the digestive tract, they need to train their gut before swallowing food for the first time; the light reflex we describe here might represent this training.

Light reflex-based pyloric opening is also important for the daily activities of sea urchins because it has been reported that phytoplankton stay at the surface range all day long but that zooplankton sink to a relatively deep region during the daytime and float to the surface at night [14], implying that sea urchin larvae need to swallow and store algae in the stomach as much as possible at night, when they are near algae. Then, when the larvae are exposed to strong sunlight, their pylori begin to open to pass the digested algae to the intestine. Simultaneously, this system, by which the pylorus tends to remain closed in the dark (Additional file 1: Fig. S3), is likely important because larvae should not pass undigested algae from the stomach to the intestine at night; therefore, it is speculated that the light-dependent pyloric opening system has been acquired and developed along with daily migration during evolution.

It is still unclear why the pylorus opened in response to light stimuli in only 40% of larvae. This might be because the light used for the experiments was simply not enough to induce pyloric opening; we used half of the number of photons as direct sunlight in our experiments, which corresponded to a depth of a few meters in the ocean. However, even when the larvae were exposed to a photon flux density corresponding to direct sunlight, the pyloric opening rate was similar to that of the experiment shown in Fig. 1b (Additional file 1: Fig. S9), leaving the question unsolved. In addition, we could not find a Go-Opsin-specific wavelength in this study, but in *Platynereis*, the blue-cyan light, which is the main component of the LED wavelength, is specific for Go-Opsin absorbance [48], suggesting that a similar range of wavelengths is effective for the pyloric opening in sea urchin larvae. This 40% opening rate increased to close to 80% after the exposure to serotonin or food algae (Figs. 2c and S3). Although future works will elucidate how food stimuli are involved in the light>serotonin>pylorus pathway, our data clearly indicates that anterior neuroectodermal serotonin is a key neurotransmitter that regulates pyloric opening and that food can stimulate the neural pathway. It is also still unclear why the pylorus opened 2 min after stimulation. It is very intriguing that the timing of pyloric opening under photoirradiation was similar with and without food stimuli. This suggests that light plays a part of the central and initial roles in the pathway of pyloric opening. In addition, because larvae have to collect the stomach contents in the pylorus to push them forward, the serotonergic system might function to control ciliary beating in the stomach for 2 min after photoreception since the 5HT_2_ receptor is likely expressed throughout the stomach. Understanding the biochemical and biophysical characteristics of Go-Opsin and the serotonergic nervous system will elucidate the regulation of the timing of pyloric opening.

Since the sea urchin genome was sequenced, the gene structure and expression patterns of Opsin family members have been reported [13, 26]. However, their functions in larval behavior have been unclear. In this study, we revealed the pathway between Go-Opsin and pyloric opening, which helps larvae pass food through the gut. Sea urchin Go-Opsin is a member of the Go(/RGR)-opsin family [21, 27] and is similar to Opsin expressed in ciliary photoreceptors in scallops and in rhabdomeric photoreceptors in *Platynereis* [48–50]. In tunicates, the ciliary photoreceptor in larvae is associated with pigmented cells [51], indicating that ciliary photoreceptors in chordates are conservatively directional [52]. Therefore, future analyses of sea urchin larval and adult ciliary photoreceptor cells will elucidate how these photoreceptors were acquired and diversified in deuterostome evolution.

## Conclusions

Our data show that the neurons at the anterior neuroectoderm regulate the function of the digestive tract in response to light stimuli in sea urchin larvae. Because the anterior neuroectoderm of sea urchin embryos/larvae is homologous to the brain region in vertebrates based on gene expression profiles and gene functions during its formation [28–30], it is suggested that the regulatory pathway between the brain and the gut was already present in the common ancestor of deuterostomes although the primitive system is not speculated yet since the vertebrates’ system, in which the vagus nerves and neural crest cells manage the brain-gut interactions, is too unique to compare with those in other systems. As the body size increased during metazoan evolution, organisms developed a sophisticated gut to digest food and obtain nutrients efficiently. Simultaneously, it is speculated that the brain-dependent gut control systems have been developed since the brain can integrate both body internal and external information and can transfer them to the digestive tract to reflect the information in regulating gut activity.

## Methods

### Animal collection and embryonic/larval culture

Adult *Hemicentrotus pulcherrimus* were collected around Shimoda Marine Research Center, University of Tsukuba, and around the Marine and Coastal Research Center, Ochanomizu University. Adult sea urchins were collected under the special harvest permission of prefectures and Japan Fishery cooperatives. Gametes were collected by the intrablastocoelic injection of 0.5 M KCl, and the embryos/larvae were cultured at 15 °C in glass beakers or plastic dishes that contained filtered natural seawater (FSW) with 50 μg/ml kanamycin. In some experiments, we fed 3.3 μl/ml SunCulture algae (*Chaetoceros calcitrans*, Marinetech, Aichi, Japan, approx. 30,000 cells/μl) to the larvae as forage.

### Photoirradiation experiments

White LED beam light (PLATA Inc., Osaka, Japan), a general LED light irradiating a broad range of visual light wavelengths, was used for the photoirradiation experiments, and the photon flux density was measured with a quantum sensor (Apogee Instruments, Logan, UT, USA). The distance between the light and sea urchin samples was adjusted to make the photon flux density to be 1000 μmol m^−2^ s^−1^. The details of the photoirradiation are shown in Additional file 1: Fig. S10. Since 6 wells in a 24-well plate can receive the LED beam light equally, all photoirradiation experiments were carried out simultaneously for as many as 6 wells. The light source was placed 6 cm above the 24-well plate to irradiate one-half the photons of sunlight (approximately 1000 μmol m^−2^ s^−1^). To obtain the sunlight-level photons (approximately 2000 μmol m^−2^ s^−1^) (Fig. S9), the light source was placed 2 cm above the 24-well plate. The samples were prepared in 900 μl or 1000 μl SW per well, and the 24-well plates were wrapped in the aluminum foil and maintained in dark incubators until use. When the experiment required neither photoirradiation nor reagent treatment, the samples were fixed with 1000 μl 7.4% formaldehyde-SW (final concentration 3.7%) within 10 s after removing the aluminum foil (Additional file 1: Fig. S10a). When the experiment required light stimulation for a certain period of time, photoirradiation was started within 10 s after removing the aluminum foil (Additional file 1: Fig. S10b). When the experiment required reagent treatment, the reagent was added within 10 s after removing the aluminum foil (Additional file 1: Fig. S10c). When the experiment required both photoirradiation and reagent treatment, we added reagents first within 5 s after removing the aluminum foil and then exposed the light to the larvae within 5 s after reagent treatment. To make the reagents or fixative diffuse throughout in the well, the plate was hand-tapped immediately after the reagents or formaldehyde-SW was added to the well.

### Microsurgery

Larvae were transferred to new 10-cm plastic dishes filled with FSW, and a part of the body of each larva was dissected under a dissecting microscope. After surgery, the larvae were transferred to new 6-cm plastic dishes filled with FSW containing 50 μg/ml kanamycin and cultured in the dark until the next day.

### Chemical treatments

Melperone hydrochloride (FUJIFILM Wako Pure Chemical Co., Osaka, Japan), ketanserin tartrate (FUJIFILM), methysergide maleate (Sigma-Aldrich, St. Louis, MO, USA), asenapine maleate (Sigma-Aldrich), and SB269970 (Sigma-Aldrich) were added to 10 μM FSW to inhibit the serotonin/monoamine pathway. The inhibitors were applied 10 s before photoirradiation. S-Nitroso-N-acetyl-D,L-penicillamine (SNAP; FUJIFILM) was used as a nitric oxide (NO) donor (final concentration of 100 μM) [23]. SNAP was applied to the larvae 5 min before observation. 3,5-Difluorophenyl-acetyl-L-alanyl-L-S-phenylglycine T-butyl ester (DAPT; Sigma-Aldrich) was used as a γ-secretase inhibitor (final 20 mM). Serotonin (Tokyo Chemical Industry, Tokyo Japan) was dissolved to distilled water just before use and applied to culture (final 10 μM). The same volume of DMSO or seawater was applied as controls for chemical treatments.

### Whole-mount in situ hybridization and immunohistochemistry

Whole-mount in situ hybridization was performed as described previously [53] with some modifications. cDNA mix from several embryonic stages was used to make RNA probes based on the *H. pulcherrimus* genome and transcriptome [20]. The samples were incubated with digoxygenin (Dig)-labeled RNA probes for *Go-Opsin* (HPU_20590) and *tryptophan 5-hydroxylase* (*tph*; HPU_21307) [22] at a final concentration of 1.2 ng/μl at 50 °C for 5 days. The Dig-labeled probes were detected with an anti-Dig POD-conjugated antibody (Roche, Basel, Switzerland) and treated with the Tyramide Signal Amplification Plus System (TSA; PerkinElmer, Waltham, MA, USA) for 8 min at room temperature (RT). When observed, the samples were incubated in MOPS buffer containing 2.5% 1,4-diazabicyclo-2-2-2-octane (DABCO; Wako Pure Chemical Co., Osaka, Japan) to prevent photobleaching.

Whole-mount immunohistochemistry was also performed as described previously [53] with some modifications. The samples were blocked with 1% skim milk in PBST for 1 h at RT and incubated with primary antibodies (dilutions: mouse anti-Synaptotagmin B (SynB) [25], 1:100; rabbit anti-Troponin-I (TnI) [31], 1:200; rabbit anti-serotonin (#S5545, RRID; AB_477522, Sigma-Aldrich), 1:1000) overnight at 4 °C. The primary antibodies were detected with a goat anti-mouse IgG Alexa Fluor Plus 555-conjugated (#A32727, RRID; AB_2633276, Thermo Fisher Scientific, Waltham, MA, USA) or a goat anti-rabbit IgG Alexa Fluor Plus 488-conjugated (#A32731, RRID; AB_2633280, Thermo Fisher Scientific) antibodies diluted 1:2000.

Double staining for SynB protein and *Go-Opsin* mRNA was performed as described previously [22] with some modifications. Samples were fixed at 4 °C for 5 h and were blocked with 1% bovine serum albumin prior to incubation with the primary antibody (1:100 dilution of mouse anti-SynB [25]) at ambient temperature for 1 h. The primary antibody was detected with a goat anti-mouse IgG HRP-conjugated antibody (#405306, RRID; AB_315009, BioLegend, San Diego, CA, USA) diluted 1:2000 and TSA treatment. After SynB detection by TSA-based immunohistochemistry, whole-mount in situ hybridization was performed to detect *Go-Opsin* under dark conditions as described above.

### Microinjection of morpholino anti-sense oligonucleotides (MO), mRNAs, and DNA

Microinjection was performed according to a previously described method [54] with injection buffer (24% glycerol, 20 mM HEPES pH 8.0 and 120 mM KCl). The morpholino (Gene Tools, Philomath, OR, USA) sequences and the in-needle concentrations in injection buffer were as follows:

Go-Opsin MO1 (0.8–1.0 mM): 5′-ATCTTCTTGAATATGCTTCCGCGCC-3′,

Go-Opsin MO2 (1.0–1.5 mM): 5′-ACGAATTCATTGTGGTCAAATCCGC-3′,

5HT_2_ MO1 (0.5–1.0 mM): 5′-GGAAAGGAACATCTCAGATCGGCCT-3′,

5HT_2_ MO2 (0.5 mM): 5′-GATGTCCTTATGGTATGTGCA-3′,

nNOS MO1 (1.0–1.5 mM): 5′-GGAAAGGAACATCTCAGATCGGCCT-3′ (previously characterized) [23], and

TPH MO (1.2 mM): 5′-ACAGAGTAGGACGTTGATGATCTAT-3′ (the specificity was checked by immunohistochemistry for serotonin (Additional file 1: Fig. S4D)).

Two non-overlapping translation-blocking morpholinos for Go-Opsin and 5HT_2_ were used to confirm the specificity of their function (Additional file 1: Fig. S6b, 7c). For negative control experiments, we injected random MO (1.0–1.5 mM, Gene Tools, Additional file 1: Fig. S6a) or injection buffer only.

The DNA construct for the putative *cis*-regulatory element of 5HT_2_ was prepared and injected as previously described [55]. Five thousand base-pairs of the genomic DNA of *H. pulcherrimus* were isolated and combined with a DNA sequence encoding Venus.

### Detection of alkaline phosphatase

To observe the stomach and intestine under clearer conditions, we detected alkaline phosphatase (AP) activity in the digestive tract. Larvae were fixed with cold 100% ethanol (− 20 °C) for 5 min and washed 3 times with PBST (PBS [Nippon Gene Co., Tokyo, Japan], 0.1% Tween-20). The samples were washed 3 times with AP buffer (100 mM Tris pH 9.5, 50 mM MgCl_2_, 100 mM NaCl, 1.0 mM levamisole, and 0.1% Tween-20), and the AP signal was detected with NBT/BCIP (Promega, Madison, WI, USA).

### Microscopy and image analysis

Live samples (Figs. 1a and S1 only) were observed under a light/fluorescence microscope (IX70, Olympus, Tokyo, Japan). The fixed and stained specimens (all samples other than those shown in Figs. 1a and S1) were observed using a light/fluorescence microscope (IX70, Olympus) and a confocal laser scanning microscope (FV10i, Olympus). All transmission images were taken with an IX70 microscope. We set the pyloric opening rate as crucial for the response to the light because the pylorus is rarely open when the larvae are maintained in the dark. All of the pyloric opening rates were judged and counted under immunohistochemically stained larvae with anti-TnI antibody as explained above (Fig. 1b, see the “Methods” section [whole-mount in situ hybridization and immunohistochemistry]). We judged that the pylorus was closed when a strong TnI signal was observed at the center of the pylorus (Fig. 1b). Each sample size (*n*) was variable because the survival rates in normal and experimental larvae were changed in each batch. The figure panels and drawings for the figures were made using Adobe Photoshop and Microsoft PowerPoint.

### Statistical analysis

No statistical methods were used to predetermine the sample sizes. All sample sizes and *p*-values are provided in the figure legends. To compare the two groups of data shown in Figs. 1b, S4e, S6a, b, and S7b, c, we used Welch’s *t*-test (two-tailed) with a significance level of 0.01 or 0.05; the *t*-values for the data in these figures were 4.6500, 8.3187, 0.26576, 3.5544, 0.42644, and 7.4837, respectively, and the degrees of freedom (d.f.) were 5.8326, 4.1031, 3.9592, 3.9315, 2.1605, and 3.427, respectively. To compare more than two groups, we used one-way ANOVA followed by Tukey’s post hoc test with a significance level of 0.01 or 0.05; the following *F* values (*F*) and d.f. were used: in Fig. 1c, *f* = 12.4959 and d.f. = 2; in Fig. 2c, *F* = 33.804 and d.f. = 2; in Fig. 2f, *F* = 143.1067 and d.f. = 2 for control and *F* = 10.1249 and d.f. = 2 for Go-Opsin MO1; in Fig. 3a, *F* = 69.4826 and d.f. = 2; in Fig. 3d, *F* = 11.6514 and d.f. = 3 for photoirradiation and *F* = 32.985 and d.f. = 5 for chemical treatments; in Fig. 4c, *F* = 6.0302 and d.f. = 3 for photoirradiation and *F* = 15.3079 and d.f. = 5 for chemical treatments; in Additional file 1: Fig. S2a, *F* = 10.2301 and d.f. = 3; in Additional file 1: Fig. S2b, *F* = 3.724 and d.f. = 3; in Additional file 1: Fig. S4a, *F* = 21.9395 and d.f. = 3; in Additional file 1: Fig. S7a, *F* = 49.6755 and d.f. = 2; in Additional file 1: Fig. S8a, *F* = 12.3052 and d.f. = 2 for the digestive tract.

## Supplementary Information

**Additional file 1: Figure S1-S10. Figure S1.** The individual timing of pyloric opening. Each bar shows the timing of pyloric opening and closing in an individual. Among 52 larvae, 8 larvae (15.4%) responded to photoirradiation. **Figure S2**. Pyloric opening rates vary based on the length of the light-dark period. a) The graph shows that the pyloric opening rate depends on the light period (room light) before exposure to darkness. The average pyloric opening rates are 1.7% (darkness only), 7.1% (no light exposure before exposure to darkness), 17.3% (10 min of light exposure), and 13.9% (12 h of light exposure). *N* = 3, n (darkness only) = 27, 65, 30, n (no light exposure before exposure to darkness) = 41, 78, 51, n (10 min of light exposure) = 34, 79, 57, n (12 h of light exposure) = 23, 35, 70. Error bars show SE. b) The graph shows that the pyloric opening rate depends on the dark period after 10 min of light exposure. The average pyloric opening rates are 2.6% (darkness only), 8.8% (30 min of darkness), 12.8% (60 min of darkness), and 19.6% (16 h of darkness). *N* = 2–4, n (darkness only) = 49, 32, n (30 min of darkness) = 40, 58, 39, 43, n (60 min of darkness) = 27, 47, 47, 69, n (16 h of darkness) = 26, 55, 79, 38. Error bars show SE. **Figure S3**. Pyloric opening rates under various conditions. The graphs show pyloric opening rates from 0 to 10 min in a 37 °C chamber (N = 3, n (0 min) = 22, 24, 42, n (1 min) = 42, 22, 39, n (2 min) = 27, 48, 42, n (3 min) = 38, 19, 40, n (4 min) = 32, 27, 39, n (5 min) = 37, 38, 26, n (6 min) = 21, 17, 58, n (7 min) = 18, 32, 59, n (8 min) = 25, 40, 43, n (9 min) = 32, 16, 34, n (10 min) = 29, 22, 49), under red light photoirradiation (N = 3, n (0 min) = 42, 82, 66, n (1 min) = 37, 66, 28, n (2 min) = 37, 82, 60, n (3 min) = 27, 20, 55, n (4 min) = 50, 77, 70, n (5 min) = 48, 32, 61, n (6 min) = 36, 79, 67, n (7 min) = 51, 43, 75, n (8 min) = 52, 47, 59, n (9 min) = 57, 43, 45, n (10 min) = 65, 56, 96), under room light photoirradiation (N = 3, n (0 min) = 31, 16, 34, n (1 min) = 20, 32, 34, n (2 min) = 42, 21, 46, n (3 min) = 28, 23, 46, n (4 min) = 37, 22, 40, n (5 min) = 29, 15, 40, n (6 min) = 54, 25, 27, n (7 min) = 23, 16, 33, n (8 min) = 37, 22, 33, n (9 min) = 31, 32, 35, n (10 min) = 38, 40, 37), and under high-intensity photoirradiation with food (*N* = 4, n (0 min) = 52, 27, 19, 20, n (1 min) = 64, 22, 12, 12, n (2 min) = 65, 25, 16,19, n (3 min) = 60, 30, 14, 22, n (4 min) = 39, 26, 15, 21, n (5 min) = 69, 29, 22, 20, n (6 min) = 52, 24, 19, 23, n (7 min) = 47, 21, 20, 13, n (8 min) = 31, 27, 15, 16, n (9 min) = 50, 18, 15, 16, n (10 min) = 45, 24, 20, 22). The average pyloric opening rates in each graph are: in the 37 °C chamber, 2.2% (0 min), 0.8% (1 min), 0.8% (2 min), 1.7% (3 min), 1.2% (4 min), 2.2% (5 min), 1.6% (6 min), 1.6% (7 min), 3.3% (8 min), 2.1% (9 min), 0.7% (10 min); under red light photoirradiation, 3.2% (0 min), 3.7% (1 min), 4.2% (2 min), 3.5% (3 min), 4.7% (4 min), 2.3% (5 min), 0.4% (6 min), 2.9% (7 min), 2.7% (8 min), 0% (9 min), 1.0% (10 min); under room light photoirradiation, 3.2% (0 min), 5.1% (1 min), 3.8% (2 min), 5.5% (3 min), 4.5% (4 min), 3.4% (5 min), 3.3% (6 min), 4.2% (7 min), 3.0% (8 min), 0% (9 min), 1.7% (10 min); under high-intensity photoirradiation with food are 9.0% (0 min), 60.9% (1 min), 83.9% (2 min), 75.4% (3 min), 61.1% (4 min), 38.8% (5 min), 25.8% (6 min), 39.7% (7 min), 19.5% (8 min), 22.6% (9 min), 21.9% (10 min). The temperature of the seawater after 10 min of heating in a 37 °C chamber was 24 °C degrees, which corresponded to the seawater temperature after 10 min of high-intensity (white LED light) photoirradiation. Sea urchin larvae responded to photoirradiation with high-intensity light (Fig. 1b) but not to heat, red light photoirradiation or and room light photoirradiation. The pyloric opening rate increased dramatically upon the addition of food. Error bars show SE. **Figure S4**. Pyloric opening rates upon the addition of serotonin and addition. a) The graph shows a serotonin concentration-dependent increase in the pyloric opening rate 2 min after the addition of serotonin under red light. The average pyloric opening rates are 4.3% (0 μM), 5.6% (0.1 μM), 33.6% (1.0 μM), and 82.5% (10 μM). *N* = 4, n (0 μM) = 53, 42, 49, 53, n (0.1 μM) = 34, 31, 60, 106, n (1.0 μM) = 26, 32, 33, 42, and n (10 μM) = 35, 48, 45, 58. Error bars show SE. b) The graph shows the change in the pyloric opening rate from 0 to 10 min after the addition of 10 μM serotonin. The average pyloric opening rate are 1.1% (0 min), 45.0% (1 min), 83.2% (2 min), 58.2% (3 min), 44.1% (4 min), 23.7% (5 min), 6.8% (6 min), 7.0% (7 min), 3.9% (8 min), 7.1% (9 min), and 4.5% (10 min). *N* = 3, n (0 min) = 31, 44, 17, n (1 min) = 46, 15, 16, n (2 min) = 22, 30, 27, n (3 min) = 29, 31, 12, n (4 min) = 33, 18, 26, n (5 min) = 24, 35, 26, n (6 min) = 32, 14, 27, n (7 min) = 41, 9, 23, n (8 min) = 15, 9, 20, n (9 min) = 18, 24, 23, and n (10 min) = 28, 25, 16. The dotted line shows the opening rate of control larvae (addition of seawater). The average pyloric opening rate are 2.9% (0 min), 4.3% (1 min), 2.4% (2 min), 4.8% (3 min), 1.9% (4 min), 3.1% (5 min), 2.3% (6 min), 5.0% (7 min), 4.8% (8 min), 0% (9 min), and 0% (10 min). *N* = 2, n (0 min) = 23, 34, n (1 min) = 26, 21, n (2 min) = 21, 21, n (3 min) = 21, 12, n (4 min) = 34, 52, n (5 min) = 45, 16, n (6 min) = 95, 28, n (7 min) = 46, 20, n (8 min) = 61, 21, n (9 min) = 54, 25, and n (10 min) = 84, 17. Error bars show SE. c) *Tryptophan hydroxylase* (*tph*; serotonin synthase) was expressed exclusively in the anterior-neuroectoderm (ANE). d) TPH morphants exhibited a loss of serotonin, but the morphology of the gut and alkaline phosphatase activity in the gut were almost normal. e) The graph shows the pyloric opening rate in control and TPH morphants under photoirradiation. The pylori of TPH morphants rarely responded to photoirradiation. The average pyloric opening rate are 20.5% (control) and 5.9% (TPH morphants). *N* = 4, n (control) = 12, 82, 63, 54, and n (TPH morphants) = 16, 12, 24, 34. Error bars show SE. Scale bars in c) and d) = 50 μm. **Figure S5.** *Go-Opsin* was expressed in nerve cells adjacent to the anterior-neuroectoderm .a) Cells in which *Go-Opsin* was co-expressed with SynB were neurons. Single optical sections of the dotted lined square in the most left panel are magnified in the other four images. The arrowhead indicates the *Go-Opsin* cell. b) The number of *Go-Opsin* cells was increased in the DAPT-treated larvae, indicating these cells are delta-positive neurons. Scale bars in a) and b) = 10 μm. **Figure S6.** Pyloric opening rates of random MO- and Go-Opsin MO2-injected larvae. a) The activity of alkaline phosphatase in the gut was invariant in random MO-injected larvae and buffer-injected larvae (Fig. 2e). The graph shows that the pyloric opening rate in random MO-injected larvae was the same as that in the control. The average pyloric opening rates are 17.5% (control; buffer-injected) and 19.0% (random MO-injected). *N* = 3, n (control; buffer-injected) = 34, 39, 41, and n (random MO-injected) = 31, 35, 26. b) The activity of alkaline phosphatase in the gut was invariant in the Go-Opsin morphants and buffer-injected larvae (Fig. 2e). The graph shows that, in contrast to the pylori of the Go-Opsin MO1-injected morphants, the pylori of Go-Opsin-MO2 morphants did not respond to photoirradiation (Fig. 2f). The average pyloric opening rates are 22.7% (control) and 3.6% (OPN3.2-MO2-injected morphants). N = 4, n (control) = 59, 35, 21, 48, and n (OPN3.2-MO2-injected morphants) = 41, 17, 12, 14. Error bars show SE. Scale bar = 50 mm. **Figure S7.** Pyloric opening rate in 5HT receptor antagonist-treated and 5HT_2_ MO2-injected larvae. a) The pyloric opening rate was dramatically reduced upon exposure to 10 μM methysergide maleate and 10 μM asenapine maleate salt (non-selective 5HT receptor antagonists). The average pyloric opening rate are 19.8% (control), 3.7% (methysergide maleate), and 1.9% (asenapine maleate). N = 4, n (control) = 69, 59, 62, 48, n (methysergide maleate) = 33, 33, 40, 44, and n (asenapine maleate) = 24, 44, 32, 30. b) However, it was not changed upon the addition of SB269970 (a 5HT_7_ antagonist). The average pyloric opening rates are 16.1% (control) and 14.5% (SB269970). *N* = 3, n (control) = 26, 40, 72, and n (SB269970) = 24, 24, 42. c) The pyloric opening rate upon photoirradiation was decreased in 5HT_2_ MO2-injected larvae, similar to the 5HT_2_ MO1-morphants (Fig. 3d). The average pyloric opening rates are 13.6% (control) and 3.7% (5HT_2_ MO2-injected morphants). N = 4, n (control) = 46, 55, 38, 37, and n (5HT_2_ MO2-injected morphants) = 18, 22, 19, 26. d) The graph shows the change in the pyloric opening rate from 0 to 10 min after the addition of 10 μM SNAP. The average pyloric opening rates are 1.2% (0 min), 21.4% (1 min), 57.4% (2 min), 62.1% (3 min), 72.8% (4 min), 82.1% (5 min), 79.0% (6 min), 69.6% (7 min), 75.0% (8 min), 83.3% (9 min), and 69.7% (10 min). N = 3, n (0 min) = 44, 28, 75, n (1 min) = 48, 15, 37, n (2 min) = 27, 34, 25, n (3 min) = 33, 19, 30, n (4 min) = 44, 22, 47, n (5 min) = 41, 28, 47, n (6 min) = 26, 27, 42, n (7 min) = 35, 18, 39, n (8 min) = 38, 25, 13, n (9 min) = 27, 12, 38, and n (10 min) = 51, 23, 32.The dotted line shows the opening rate of the DMSO control. The average pyloric opening rates are 0.6% (0 min), 2.9% (1 min), 4.7% (2 min), 6.3% (3 min), 6.3% (4 min), 5.4% (5 min), 2.9% (6 min), 5.6% (7 min), 2.0% (8 min), 2.0% (9 min), and 2.9% (10 min). N = 3, n (0 min) = 38, 24, 55, n (1 min) = 36, 14, 17, n (2 min) = 26, 19, 20, n (3 min) = 49, 23, 26, n (4 min) =47, 19, 25, n (5 min) = 73, 18, 32, n (6 min) = 57, 32, 72, n (7 min) =54, 19, 56, n (8 min) = 63, 19, 45, n (9 min) = 43, 16, 50, and n (10 min) = 61, 23, 23. Error bars show SE. **Figure S8**. The localization of Venus driven by the putative *cis*-regulatory element of the 5HT_2_ receptor. a) The graph shows the ratio of the location of Venus in the digestive tract and outside the digestive tract in the larvae injected with the 5HT_2_ *cis*-regulatory element fused with the Venus sequence. The average Venus expression rates are 80.6% (stomach), 5.8% (intestine), 24.2% (esophagus/mouth), 70.0% (pigment cells), 55.1% (tip of arms and posterior), and 10.8% (others). N = 3, *n* = 23, 22, 25. Error bars show SE. b) Venus signals were observed outside the digestive tract. Scale bars in b) = 20 μm. **Figure S9**. Pyloric opening rate at photon flux density corresponding to sunlight. The graph shows the pyloric opening rate upon exposure to LED light with a photon flux density that corresponded to sunlight. The average pyloric opening rates are 1.9% (0 min), 17.8% (1 min), 22.4% (2 min), 8.3% (3 min), 2.0% (4 min), 1.3% (5 min), 4.0% (6 min), 2.8% (7 min), 0.9% (8 min), 6.8% (9 min), and 0.7% (10 min). N = 3, n (0 min) = 35, 27, 21, n (1 min) = 73, 28, 19, n (2 min) = 44, 24, 18, n (3 min) = 48, 16, 9, n (4 min) = 45, 21, 27, n (5 min) = 27, 11, 25, n (6 min) = 68, 22, 27, n (7 min) =43, 26, 22, n (8 min) = 38, 21, 27, n (9 min) = 33, 18, 26, and n (10 min) = 46, 11, 24. Error bars show SE. **Figure S10.** Schematic images of the methods for larvae fixation with/without photoirradiation. White LED beam light, a general LED light irradiating a broad range of visual light wavelengths, was used for these experiments, and the photon flux density was measured with a quantum sensor. All of photoirradiation experiments were performed in 24-well plates. a) When we fixed samples without photoirradiation, we applied 2x fixative directly to the well immediately after removing the aluminum foil. b) When we fixed the light-responded samples, the plate was photoirradiated immediately after the aluminum foil was removed, and then the larvae were fixed in the well. c) When we treated the larvae with chemical reagents, we applied 100 μl of 10x concentrated reagents to the larvae immediately after removing the aluminum foil. Then, we fixed them. d) When we need the chemical reagent- and photoirradiated-larvae, we treated the samples with reagents and photoirradiation within total 10 s. Then, we fixed the treated larvae.

**Additional file 2: Movie 1.** The pylorus of a sea urchin larva opens in response to the photoirradiation. The movie shows the pyloric opening after the strong photoirradiation from the dark condition. The speed of the movie is 10x of the normal speed.

## Data Availability

All data generated or analyzed during this study are included in this published article and its supplementary information files. The sequence data used for this study are deposited in DDBJ (http://www.ddbj.nig.ac.jp/; BioProject Accession PRJDB6441). The accessions of genome and transcriptome are BEXV01000001-01016251 and IACU01000001-IACU01124330, respectively. They can also be found in the genome database of *Hemicentrotus pulcherrimus*, HpBase (http://cell-innovation.nig.ac.jp/Hpul/) [20].

## Abbreviations

ANE: Anterior neuroectoderm
SynB: Synaptotagmin B
TPH: Tryptophan 5-hydroxylase
DAPI: 4′,6-Diamidino-2-phenylindole
SNAP: S-Nitroso-N-acetyl-DL-penicillamine
AP: Alkaline phosphatase
PBS: Phosphate-buffered saline
MO: Morpholino oligonucleotide
RT: Room temperature
HRP: Horseradish peroxidase
