# Supplementary material for: Sea urchin larvae utilize light for regulating the pyloric opening
Source: BMC Biol. 2021 Apr 6;19:64. doi: 10.1186/s12915-021-00999-1 (PMC8022552; doi:10.1186/s12915-021-00999-1)
Supplement: Supplementary file 1 — Additional file 1: Figure S1-S10. Figure S1. The individual timing of pyloric opening. Each bar shows the timing of pyloric opening and closing in an individual. Among 52 larvae, 8 larvae (15.4%) responded to photoirradiation. Figure S2. Pyloric opening rates vary based on the length of the light-dark period. a) The graph shows that the pyloric opening rate depends on the light period (room light) before exposure to darkness. The average pyloric opening rates are 1.7% (darkness only), 7.1% (no light exposure before exposure to darkness), 17.3% (10 min of light exposure), and 13.9% (12 h of light exposure). N = 3, n (darkness only) = 27, 65, 30, n (no light exposure before exposure to darkness) = 41, 78, 51, n (10 min of light exposure) = 34, 79, 57, n (12 h of light exposure) = 23, 35, 70. Error bars show SE. b) The graph shows that the pyloric opening rate depends on the dark period after 10 min of light exposure. The average pyloric opening rates are 2.6% (darkness only), 8.8% (30 min of darkness), 12.8% (60 min of darkness), and 19.6% (16 h of darkness). N = 2–4, n (darkness only) = 49, 32, n (30 min of darkness) = 40, 58, 39, 43, n (60 min of darkness) = 27, 47, 47, 69, n (16 h of darkness) = 26, 55, 79, 38. Error bars show SE. Figure S3. Pyloric opening rates under various conditions. The graphs show pyloric opening rates from 0 to 10 min in a 37 °C chamber (N = 3, n (0 min) = 22, 24, 42, n (1 min) = 42, 22, 39, n (2 min) = 27, 48, 42, n (3 min) = 38, 19, 40, n (4 min) = 32, 27, 39, n (5 min) = 37, 38, 26, n (6 min) = 21, 17, 58, n (7 min) = 18, 32, 59, n (8 min) = 25, 40, 43, n (9 min) = 32, 16, 34, n (10 min) = 29, 22, 49), under red light photoirradiation (N = 3, n (0 min) = 42, 82, 66, n (1 min) = 37, 66, 28, n (2 min) = 37, 82, 60, n (3 min) = 27, 20, 55, n (4 min) = 50, 77, 70, n (5 min) = 48, 32, 61, n (6 min) = 36, 79, 67, n (7 min) = 51, 43, 75, n (8 min) = 52, 47, 59, n (9 min) = 57, 43, 45, n (10 min) = 65, 56, 96), under room light photoirradiation [file 12915_2021_999_MOESM1_ESM.docx]

Sea urchin larvae utilize light for regulating the pyloric opening

Junko Yaguchi^1^ and Shunsuke Yaguchi^1,2^*

^1^Shimoda Marine Research Center, University of Tsukuba, 5-10-1 Shimoda, Shizuoka, 415-0025 Japan

^2^PRESTO, JST, 4-1-8 Honcho, Kawaguchi, 332-0012 Japan

Key words: sea urchin, opsin, serotonin, nitric oxide, gut

*Corresponding author; Shunsuke Yaguchi, Shimoda Marine Research Center, University of Tsukuba, 5-10-1 Shimoda, Shizuoka, 415-0025 Japan

Phone; +81-558-22-1317

Fax; +81-558-22-0346

E-mail; yag@shimoda.tsukuba.ac.jp

**Additional File 1**

**Fig.S1-S10.**

**
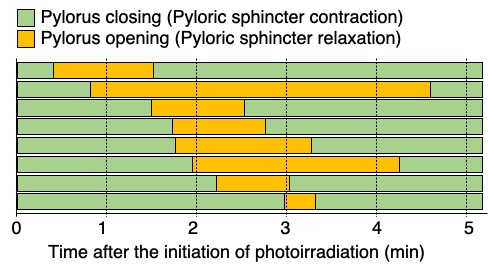
**

**Figure S1.** **The individual timing of pyloric opening.**

**
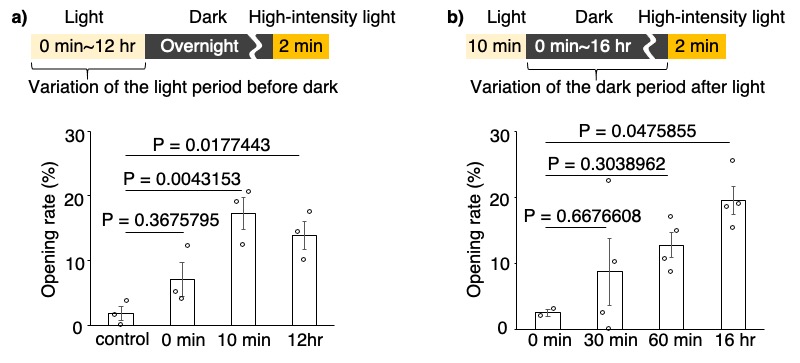
**

**Figure S2. Pyloric opening rates vary based on the length of the light-dark period.**


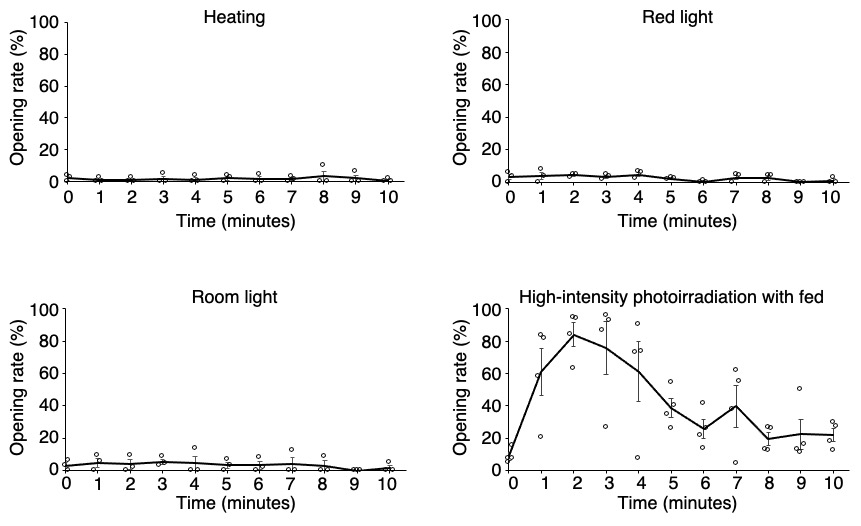


**Figure S3. Pyloric opening rates under various conditions.**


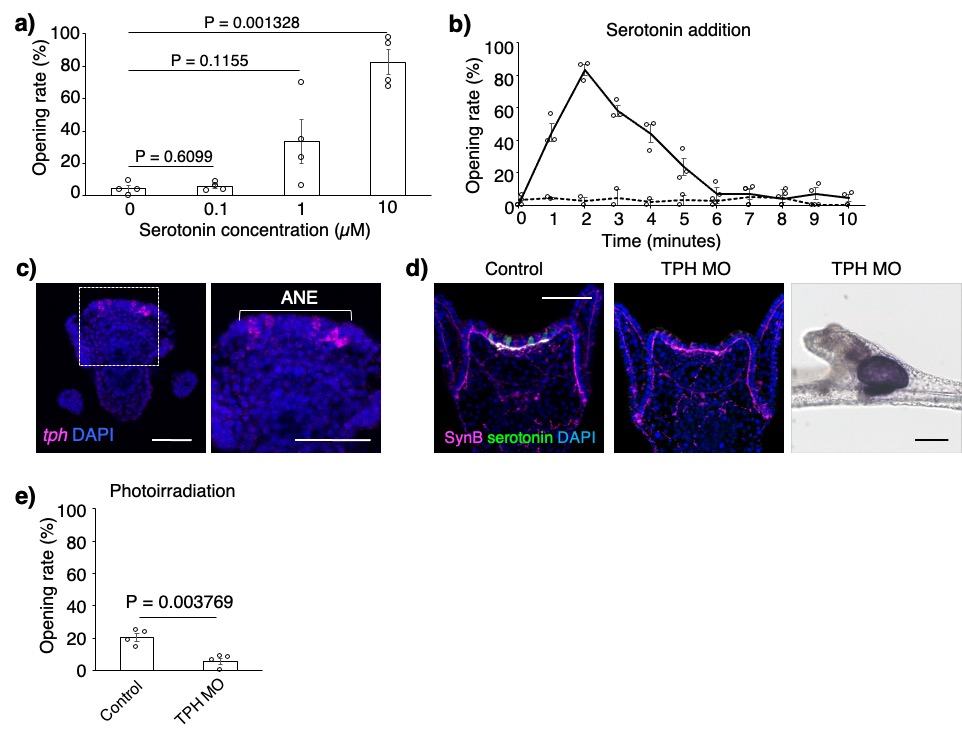


**Figure S4. Pyloric opening rates upon the addition of serotonin and addition.**


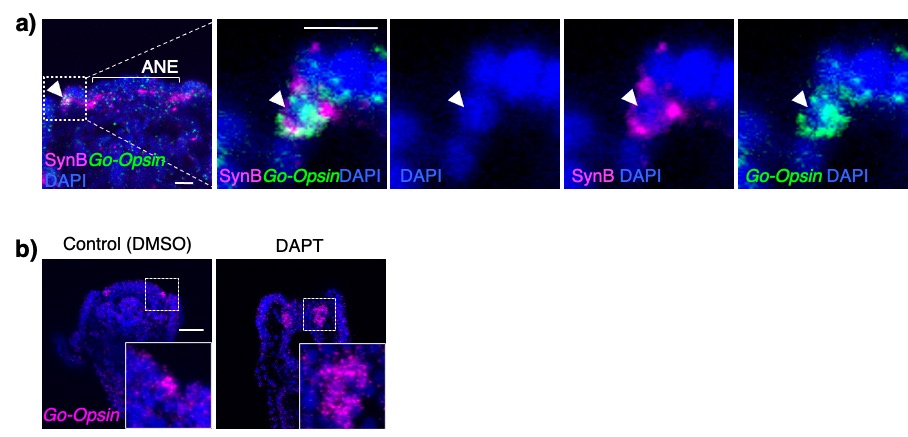


**Figure S5. *Go-Opsin* was expressed in nerve cells adjacent to the anterior-neuroectoderm .**


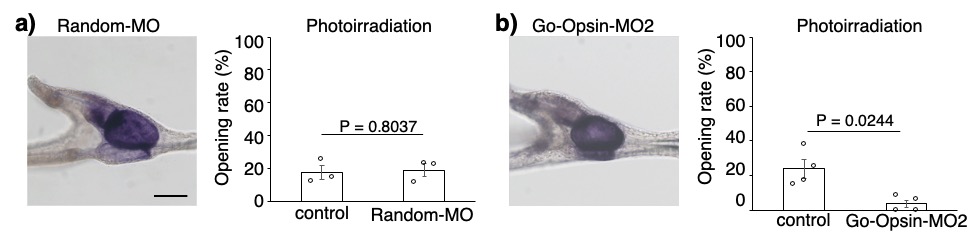


**Figure S6. Pyloric opening rates of random MO- and Go-Opsin MO2-injected larvae.**


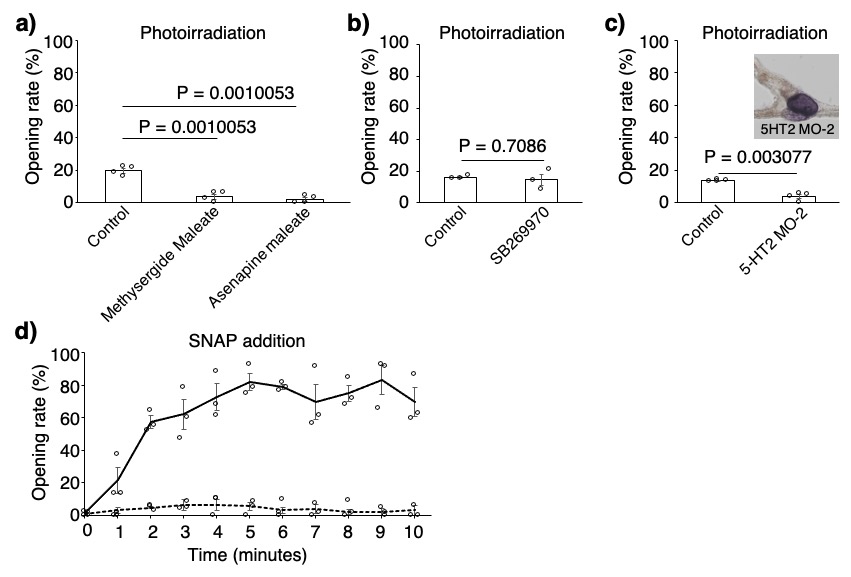


**Figure S7. Pyloric opening rate in 5HT receptor antagonist-treated and 5HT_2_ MO2-injected larvae.**


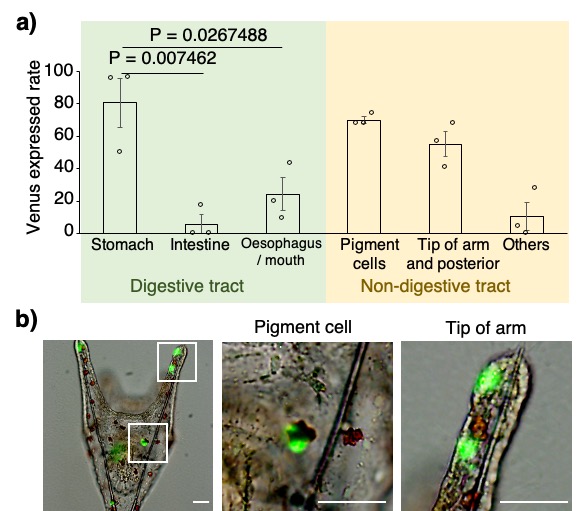


**Figure S8. The localization of Venus driven by the putative *cis*-regulatory element of the 5HT_2_ receptor.**


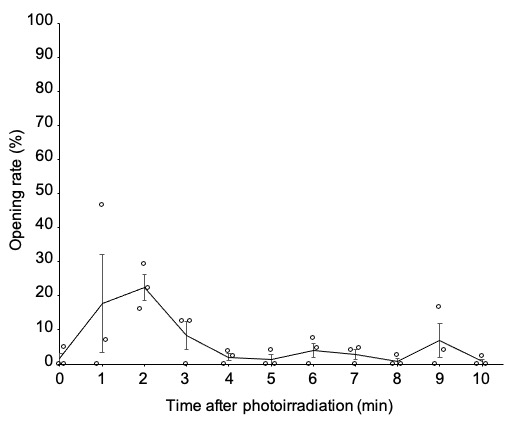


**Figure S9. Pyloric opening rate at photon flux density corresponding to sunlight.**


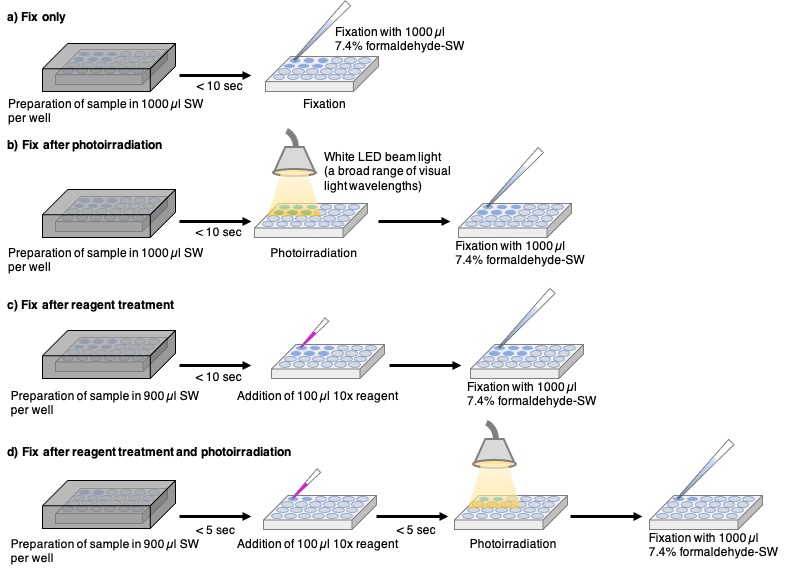


**Figure S10. Schematic images of the methods for larvae fixation with/without photoirradiation.**
